# Auditory-vocal coupling in the naked mole-rat, a mammal with poor auditory thresholds

**DOI:** 10.1007/s00359-018-1287-8

**Published:** 2018-09-19

**Authors:** Kazuo Okanoya, Shigeto Yosida, Catherine M. Barone, Daniel T. Applegate, Elizabeth F. Brittan-Powell, Robert J. Dooling, Thomas J. Park

**Affiliations:** 1 Department of Life Sciences, The University of Tokyo, Tokyo, Japan; 2 Department of Cognitive and Information Sciences, Chiba University, Chiba, Japan; 3 Department of Biological Sciences, University of Illinois, Chicago, IL USA; 4 Department of Psychology, University of Maryland, College Park, MD USA

**Keywords:** Auditory brainstem response, Vocal communication, Auditory threshold, Alarm call, Signature call

## Abstract

Naked mole-rats are extremely social and extremely vocal rodents, displaying a wide range of functionally distinct call types and vocalizing almost continuously. Their vocalizations are low frequency, and a behavioral audiogram has shown that naked mole-rats, like other subterranean mammals, hear only low frequencies. Hence, the frequency range of their hearing and vocalizations appears to be well matched. However, even at low frequencies, naked mole-rats show very poor auditory thresholds, suggesting vocal communication may be effective only over short distances. However, in a tunnel environment where low frequency sounds propagate well and background noise is low, it may be that vocalizations travel considerable distances at suprathreshold intensities. Here, we confirmed hearing sensitivity using the auditory brainstem response; we characterized signature and alarm calls in intensity and frequency domains and we measured the effects of propagation through tubes with the diameter of naked mole-rat tunnels. Signature calls—used for intimate communication—could travel 3–8 m at suprathreshold intensities, and alarm calls (lower frequency and higher intensity), could travel up to 15 m. Despite this species’ poor hearing sensitivity, the naked mole-rat displays a functional, coupled auditory-vocal communication system—a hallmark principle of acoustic communication systems across taxa.

## Introduction

**Fig. 1:**
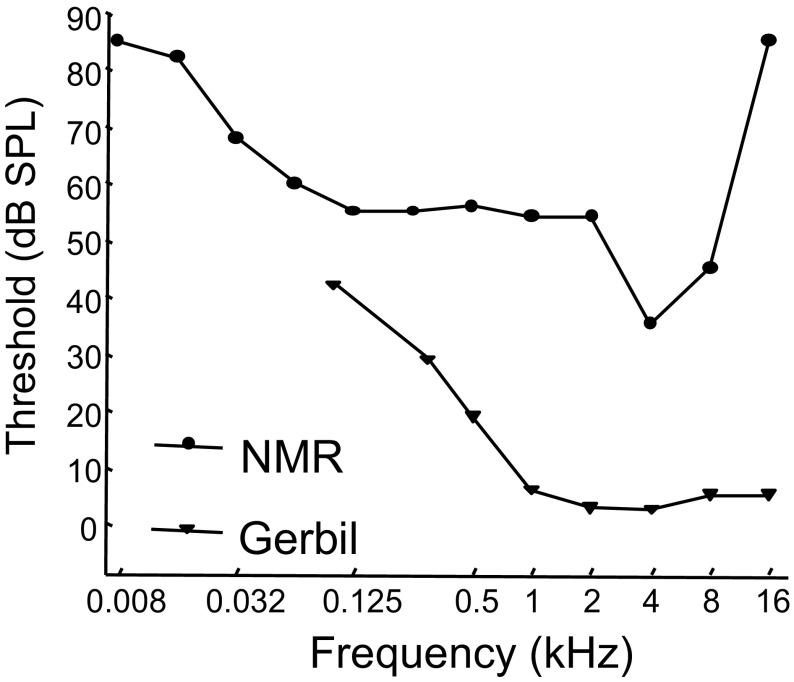
Audiograms for naked mole-rats and gerbils. Behavioral audiogram for the naked mole-rat (closed circle) re-drawn from Heffner and Heffner (1993; data points for lowest two frequencies tested not shown). A behavioral audiogram for the gerbil (Ryan 1976; open triangle) is shown for comparison. *NMR* naked mole-rat

Naked mole-rats are extremely social rodents. They live in large colonies that can include hundreds of individuals, with usually only one breeding female and one to three breeding males. The remaining adults are divided into at least two non-breeding social castes: soldiers and housekeepers (Jarvis 1981, 1991; Lacey and Sherman 1991). Naked mole-rats are also extremely vocal rodents, both in terms of how often they vocalize and the number of different call types that they produce. Within captive colonies, there is a continuous chatter of vocalizations, from which Pepper et al. (1991) identified 17 different call types associated with a variety of behavioral contexts. The naked mole-rats’ high rate of vocalizing and their extensive vocal repertoire suggest that these animals rely heavily on auditory-vocal communication—a trait that is usually associated with good hearing capacity. However, a behavioral assay of their hearing (Heffner and Heffner 1993) revealed that, like other fossorial mammals, naked mole-rats have markedly higher auditory thresholds compared to non-fossorial, low frequency-hearing mammals (Brückmann and Burda 1997). Figure 1 shows the behavioral audiogram for naked mole-rats, and for comparison, an audiogram from gerbils (low frequency specialists). Note that thresholds for the naked mole-rats are substantially higher than those of the gerbils. Hence, there is an apparent incongruity for vocal communication in naked mole-rats; vocal signaling appears to be important to these animals, yet they also appear to have relatively poor hearing sensitivity.

This apparent incongruity is particularly bothersome in light of the overwhelming evidence that exists for the co-evolution of coupled auditory and vocal systems across animal taxa (e.g., frog, cricket, fish, birds, bats; Ryan 1986; Gentner and Margoliash 2003; Sisneros and Bass 2003; Woolley and Moore 2011). Considering this potent evolutionary theme, one possible explanation pertaining to the naked mole-rat is that high auditory thresholds do not impair reception of species-specific vocal signals within the naked mole-rats’ tunnel umwelt where low frequency sounds should propagate well, and environmental background noise should be minimal. Hence, theoretically, the naked mole-rat auditory system may be capable of receiving species-specific vocal signals at suprathreshold intensities over considerable distances.

We already know that hearing and vocal production are coupled within the spectral domain, both being in the low frequency range (Pepper et al. 1991; Heffner and Heffner 1993). What remains to be determined for the naked mole-rats is whether or not calls are produced with enough energy to propagate an appreciable distance through the tunnels at suprathreshold intensities. If not, vocal communication for this species may be limited to only relatively short distances. To address this issue, we first derived a physiological measure of hearing capacity to confirm the previous behavioral measures. We then characterized the acoustic properties of two naked mole-rat calls types (alarm calls and signature calls), focusing on both spectral content and on the intensity at which calls are produced. Finally, we measured the propagation properties of these calls traveling through tubes with the diameter of naked mole-rat tunnels.

## Materials and methods

The original research reported herein was performed under guidelines established by the Institutional Animal Care and Use Committees at the University of Illinois at Chicago, the University of Maryland, Chiba University, and the RIKEN Brain Science Institute.

### Auditory brainstem response

We measured auditory brainstem responses from four naked mole-rats (NMR, *Heterocephalus glaber*) and two mongolian gerbils (*Meriones unguiculatus*). The naked mole-rats included one breeding female, one breeding male, and two non-breeding adult females. The ages of these animals were 6.3 years, 3.4 years, 1 year, and 6 years, respectively. Captive naked mole-rats can live to be over 30 years old, and we consider 1–6 year olds to be young adults (Buffenstein et al. 2012). The gerbils were between 2 and 8 months, also considered to be young adults (mongolian gerbils can live to be 5 years old).

All animals were sedated with subcutaneous injection of Ketamine (35–50 mg/kg) and Xylazine (8 mg/kg) prior to electrode placement. Animals remained relatively motionless for up to 75 min. Body temperature was maintained at 30 ± 0.5 °C (NMR) and 37 ± 0.5 °C (gerbils) with a heating pad and monitored with a thermistor probe (Frederick Haer and Co., Model 40-90). Note that naked mole-rats are poikilotherms, and in nature, their body temperature would reflect the ambient temperature in their tunnels which is usually about 30 °C (Bennett and Faulkes 2000).

Standard platinum alloy, subdermal needle electrodes, (Grass F-E2; West Warwick, RI, USA) were placed just under the skin high at the vertex (active), directly behind the right ear canal (the ear ipsilateral to the speaker, reference) and behind the canal of the ear contralateral to stimulation (ground). Shielded electrode leads were twisted together to reduce electrical noise through common mode rejection.

The stimulus presentation, ABR acquisition, equipment control, and data management were coordinated using a Tucker-Davis Technologies (TDT, Gainesville, FL, USA) modular rack-mount system controlled by an optical cable-linked 350-MHz Pentium PC containing a TDT AP2 Digital Signal Process board and running TDT ‘BIOSIG’ software. Sound stimuli were generated using TDT ‘SIGGEN’ software and fed through a DA1 digital-analog converter, a PA4 programmable attenuator, and a HB6 transducer which directly drove the JBL Professional Series speaker (Model 2105H, James B Lansing Sounds Inc.). The electrodes were connected to the TDT HS4 Headstage that amplifies and digitizes the signal before sending it over fiber optic cables to the TDT DB4 Digital Biological Amplifier. This amplifier allows additional filtering and gain to be added. A TDT TG6 timing generator synchronized the A/D and D/A conversion.

Stimulus intensities were measured in the free field by placing the ½-in. microphone of a sound level meter (System 824; Larson Davis, Inc. Provo, UT, USA) at the approximate position of the animal’s ear (30 cm from speaker). Tones were played continuously using the TDT BIOSIG program and measured using the fast A-weighted scale on the SLM. For values below 1 kHz, the A-filter values were corrected for the filter. To determine the intensity of the short duration click, we used the peak equivalent SPL of the click. This was determined using an oscilloscope and noting the peak-to-peak voltage of the click. A test tone, e.g., a 1 kHz tone, was played and adjusted until the peak-to-peak voltage was the same as it was for the click. The SPL required to match the amplitude of the click, as indicated at the sound level meter, was the peak equivalent SPL (dB pSPL) of the click stimulus.

### Stimuli

Subjects were presented with multiple intensity stimulus trains that varied in frequency and intensity (see Brittan-Powell et al. 2002, 2005; Brittan-Powell and Dooling 2004; Wright et al. 2004). Each train consisted of 9 tone bursts or clicks. Stimulus trains were presented at a rate of 4/s and progressively increased in intensity. The click trains consisted of rectangular-pulse broadband clicks were 0.1 ms (100 µs) in duration with 25 ms inter stimulus interval (ISI). Each individual tone burst was 5 ms in duration (1 ms rise/fall COS2) with a 20 ms ISI. Short rise times were used because they have less of an effect on ABR latency and wave morphology (Hecox et al. 1976; Kodera et al. 1977, 1979), and nonlinear gating methods, such as COS2, provide narrowband amplitude spectrum and considerable reduction of amplitudes of side lobes that can detract from frequency specificity (Robier et al. 1992). The tone bursts used were 0.25, 0.5, 1, 2, 4, 8 and 10 kHz, with intensities spanning a 40-dB range in ascending order of 5 dB steps (e.g., started at 70 dB and increasing to 110 dB or starting at 30 dB and increasing to 70 dB). Tone burst spectra were generated using 1024-point fast Fourier transform (FFT) and showed all harmonics were at least 20 dB down from the peak of the frequency of interest.

Each ABR represents the average response of 600 stimulus presentations (300 averages for each polarity/phase were added together to cancel the cochlear microphonic), sampled at 20 kHz for 235 ms following onset of the stimulus. The biological signal was amplified (× 100K) and notch filtered at 60 Hz with the DB4 Digital Biological Amplifier during collection; the averaged signal was bandpass filtered below 30 Hz and above 3000 Hz after collection using BIOSIG.

**Fig. 2:**
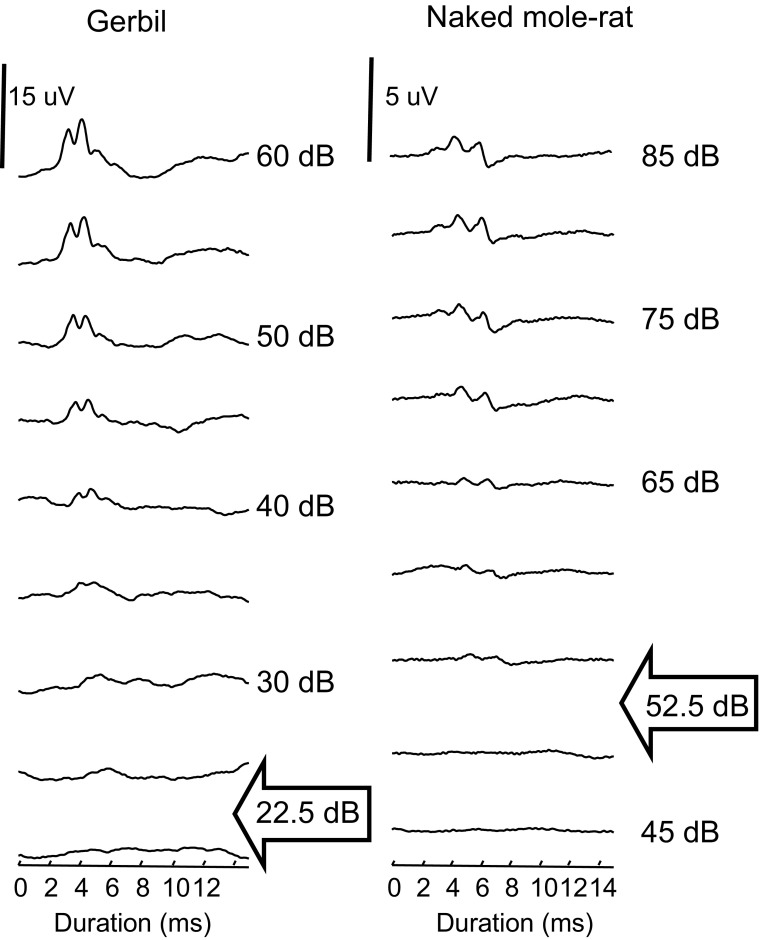
ABR waveforms evoked by a 2 kHz tone train presented at different intensities for a gerbil and a naked mole-rat. Arrows indicate thresholds for this frequency, 22.5 dB for the gerbil and 52.5 dB for the naked mole-rat. Due to the differences in thresholds, the range of intensities shown are different for the two animals. Note that the scale bars (uV) also differ between species because ABR waveform amplitudes for the naked mole-rat were smaller in general compared to the gerbil. This species difference was consistent across all animals tested

Thresholds were estimated using the visual detection method (Brittan-Powell and Dooling 2004; Brittan-Powell et al. 2005): the lowest intensity at which a response could be detected visually on the trace, regardless of wave, or 2.5 dB below the lowest intensity that elicited a measurable response (examples will be given below in Fig. 2 of the “Results”).

### Analysis of vocalizations

Recordings were made in a wooden box (45 × 30 × 30 cm) lined with acoustic foam panels (thickness: 2 cm) in a sound attenuating chamber. We placed a condenser microphone (SONY ECM-MS957) 20 cm above the floor of the recording box. The microphone was connected to a Windows compatible PC through a preamplifier and a sound card (ONKYO SE-U77). Recordings to the hard disk were carried out via Avisoft-SASlab Pro (Avisoft Bioacoustics, Germany) set at a 44.1-kHz sampling rate and a 16-bit resolution, and the recorded sound was stored as a wave file. The animals we recorded from were all 9–15 months old and similarly sized, and we presumed that they were all in the same caste (i.e. “workers” not “soldiers”). Signature calls were recorded by gently pushing the animal’s back (Yosida et al. 2007; Yosida and Okanoya 2009). Alarm calls were recorded by grabbing the animal’s tail. At least five instances of each call were recorded per animal. Spectrograms of all calls were produced with a 240 Hz analysis bandwidth using Avisoft.

### Sound pressure measurements

While recording these vocalizations, the microphone of the sound level meter (RION) was placed 20 cm above the floor of the recording box and real-time measurement of the sound intensity of each recorded call was performed. The sound level meter was set at an A-weighted scale and fast integration time. Since the animal moved about the floor of the recording chamber, exact distance between the animal and the tip of the microphone could not be determined. We estimated the average distance to be 24 cm. Based on this, the sound pressure level at 12 cm from the animal’s mouth was estimated to be 6 dB higher than that actually recorded.

### Tunnel diameter and transmission characteristics

To assess how sound propagation through a naked mole-rat tunnel would affect the characteristics of calls, we first had to derive an estimate of naked mole-rat tunnel diameter. We accomplished this by allowing naked mole-rats to dig tunnels within a 1 cubic meter tub of compacted, sterilized soil for 48 h. For this procedure, we used a small colony of animals consisting of 6 adults. We attached their home cage system—a series of mouse cages connected by PVC pipes (Artwohl et al. 2002)—to the tub of soil. During the 48 h access to the soil, the naked mole-rats excavated approximately 5 m of tunnel. We made plaster casts of the tunnels and took diameter measures from the casts. We measured tunnel diameter at 15 cm intervals and found that the average diameter was 4.03 cm (35 measurements, SD = 0.884 cm). Based on those data, and published observations from tunnels excavated in the field (Jarvis and Bennett 1991), we used pipe with a similar diameter (4 cm) to assess how calls would propagate through tunnels.

We assessed the transmission characteristics of 4 cm diameter pipe by playing different frequency tones (0.5, 3.1, and 10.6 kHz), and recorded calls through a variety of pipe lengths (60, 240, 360, 460, and 480 cm) and two inner surface textures: plain PVC pipe and pipe lined with soft rice paper. Tones were generated with the same equipment as those for testing ABRs. For assessed transmission characteristics, we used tones with a duration of 200 ms with 10 ms rise/fall times. Two of the frequencies we chose were similar to the fundamental frequencies of alarm calls (0.5 kHz) and signature calls (3.1 kHz). 10.6 kHz was chosen as a third frequency so that we would have data for a frequency at the upper limit of the naked mole-rat’s hearing ability (Heffner and Heffner 1993). Tones and calls were played with a peak amplitude of 80 dB. The plain PVC pipe is relatively non-sound absorbing so we also measured transmission characteristics through pipe lined with soft rice paper to simulate a relatively greater absorbing environment. Note that in burrows, audible sounds appear to propagate much more through the air in the burrow than through the soil (Narins et al. 1997).

## Results

Here, we report on the hearing capacity of naked mole-rats, the characteristics of their alarm and signature calls, and the propagation characteristics of these calls through tubes with a diameter based on naked mole-rat tunnels.

### Auditory brainstem response (ABR)

We measured the ABR from four naked mole-rats and two gerbils. Gerbils were chosen as a comparison species because there is an abundance of both ABR and behaviorally derived audiogram data in the literature for this species (e.g., McFadden et al. 1996).

ABR waveforms were similar for naked mole-rats and gerbils except that the amplitude of the naked mole-rat response was only about 25% that of the gerbil. Figure 2 shows ABR waveforms evoked by different intensities from a naked mole-rat and a gerbil tested with 2 kHz tones. Visual examination of the waveforms showed 2–3 prominent peaks that occurred within the first 8 ms after sound reached the animal’s external ear canal. As with all animals tested to date (e.g., Hecox and Galambos 1974; Starr and Achor 1975; Picton et al. 1976), increasing the intensity of stimulation, caused two major changes: latencies to all waves decreased, and amplitudes of all waves increased.

As mentioned in the “Materials and methods” section above, thresholds for a given frequency were estimated visually from the waveforms. For the data in Fig. 2, we estimated a threshold for the gerbil to be 22.5 dB, and we estimated threshold for the naked mole-rat to be 52.5 dB (both indicated by arrows).

**Fig. 3:**
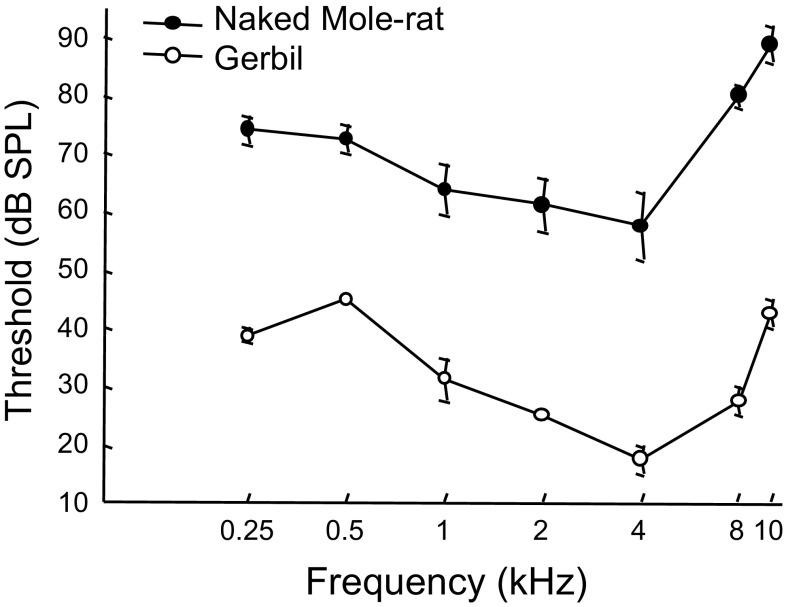
ABR-derived audiograms for four naked mole-rats and two gerbils. Error bars are s.e.m.

We used ABR data from across frequencies and intensities to construct audiogram curves, which are shown in Fig. 3. Each point on an audiogram curve corresponds to a threshold response for a particular frequency. For example, the 0.25 kHz tone evoked a threshold response from the naked mole-rats at an average intensity of 75 dB, and from the gerbils at an average intensity of 40 dB.

The audiograms from both species had a characteristic U-shaped function, but the naked mole-rats had consistently higher thresholds compared to the gerbils. On average, thresholds for the naked mole-rats were between 25 and 50 dB higher than those of the gerbils, which is consistent with the differences observed in the behavioral audiograms shown in Fig. 1.

Comparison to the behaviorally derived audiograms presented in Fig. 1 shows that the ABR derived curves are elevated by about 15–25 dB for both naked mole-rats and gerbils. This overall difference between behaviorally and ABR derived audiograms is consistent with previous data comparing the two techniques (e.g., Gorga et al. 1988; Werner et al. 1993; Brittan-Powell et al. 2002). Since the ABR is an onset response, much of the difference may be attributed to differences in temporal integration (Brittan-Powell et al. 2002). Given the consistent difference between behaviorally and ABR derived audiograms across a wide range of species, we interpret our present naked mole-rat ABR audiogram to be in good agreement with the previously reported behavioral audiogram.

### Analysis of naked mole-rat vocalizations

We recorded five alarm calls and five signature calls from each of five individual naked mole-rats. We focused on these two call types because they are acoustically distinct and because they are associated with very different behavioral contexts (Pepper et al. 1991). The alarm call (also referred to as the “grunt” call) functions as a colony defense call (Pepper et al. 1991). The signature call (also referred to as the “soft chirp” call) is the most common vocalization, and appears to function as a short distance, close contact call, usually emitted when animals touch one another (Pepper et al. 1991; Yosida et al. 2007). Within individuals these calls are emitted with a high degree of reproducibility. Across individuals they vary widely in duration but remain very similar in spectral content and intensity (Yosida and Okanoya 2009).

**Fig. 4:**
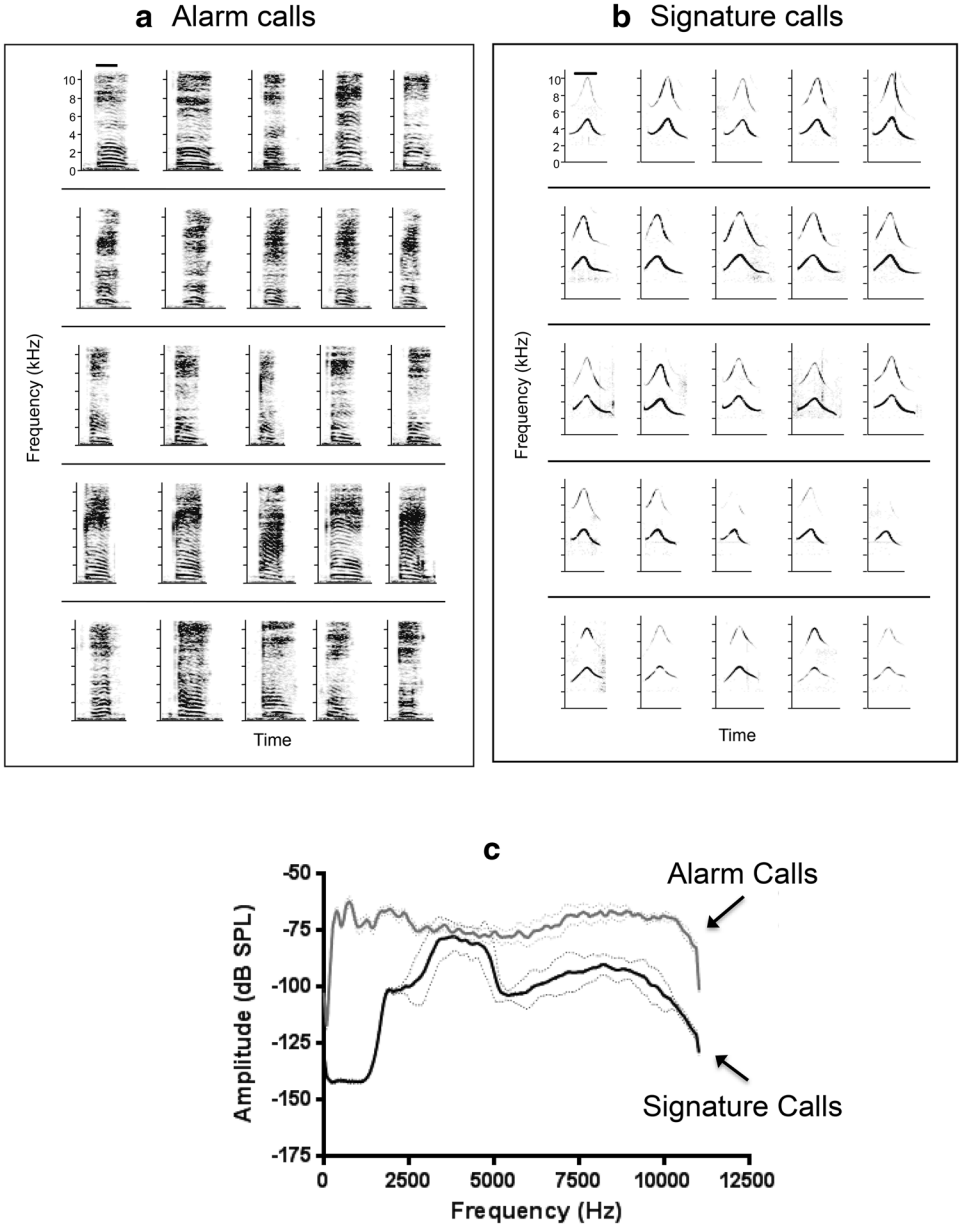
shows representative spectrograms and average power spectra from alarm calls and signature calls. **a** Alarm calls from five different individual naked mole-rats, five calls each (each row is from one animal). The scale bar in the upper left is 100 ms. **b** Signature calls from five different individual naked mole-rats, five calls each (each row is from one animal). The scale bar in the upper left is 100 ms. **c** Average power spectra from five animals, five calls each for alarm and signature calls

Spectrograms of alarm and signature calls from different individuals are displayed in Fig. 4. For the alarm calls (Fig. 4a), the fundamental frequency was about 300 Hz. Each call had a fundamental plus 4–5 harmonics, with call length ranging from 100 to 150 ms. Alarm calls were produced at an average intensity of 85.9 dB (grand average of five animals, five calls each, SD = 0.93), measured at 20 cm from the animal. For the signature calls (Fig. 4b), the fundamental frequency was between 3 and 5 kHz with a characteristics frequency modulation that first went up and then down. Each call had a fundamental plus 1–3 harmonics, with call length ranging from 100 to 200 ms. The signature calls were produced at an average intensity of 63.4 dB (grand average of five calls from five animals, SD = 0.60) measured 20 cm from the animal. To summarize the characteristics of the alarm and signature calls across individuals, we calculated the average power spectrum from five animals, five calls each which is presented in Fig. 4c.

### Transmission characteristics of simulated tunnels

Naked mole-rats in our laboratory dug tunnels with an average diameter of 4.03 cm (35 measurements over 5 m of tunnel, SD = 0.884 cm), which corresponds closely to measurements made from excavated tunnels in the field (Jarvis and Bennett 1991). Attenuation level of a sound in a tunnel would depend on at least two factors. First, since the tunnel limits the diffusion of sound energy proportional to the square of the distance from the source, an ideal tunnel should reduce the amount of attenuation that follows the inverse square law. Second, since the surface of the tunnel would reflect and/or absorb the sound energy, the physical property of the sound would give excess attenuation. Because of the complexity of this acoustics, we can not theoretically predict attenuation levels of the calls broadcast in the tunnel of naked mole-rats.

**Fig. 5:**
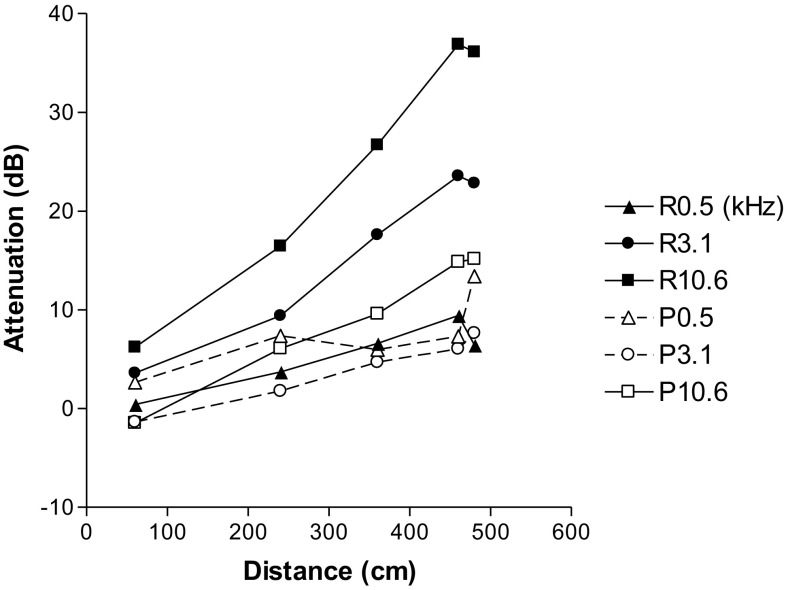
Attenuation through pipes as a function of distance and frequency. Tones with frequencies of 0.5, 3.1, and 10.6 kHz were played through plain PVC pipe (dashed lines with open symbols, “P” in the legend) or played through PVC pipe lined with soft rice paper (solid lines with solid symbols, “R” in the legend)

To measure actual attenuation of the sound in a simulated naked mole-rat tunnel, we played tones and calls through a 4 cm PVC pipe using a variety of pipe lengths, and two inner surface textures: plain PVC pipe and pipe lined with soft, sound absorbent rice paper. While the pipe is obviously an idealized tube compared to actual mole-rat tunnels, both retain the principles of a tube structure. The curves in Fig. 5 show how the pipe affected the intensity of tones as a function of distance and tone frequency for three representative frequencies. The set of dashed curves were measured with the plain pipe, and the set of solid curves were measured with the pipe lined with soft rice paper. In both cases, higher frequencies showed greater attenuation over distance than lower frequencies, as was expected from the low-pass filter properties of tubes.

Tones of 0.5 kHz, roughly the frequency of peak energy for the alarm calls, were attenuated by about 10 dB over 5 m through both the plain PVC pipe (dashed curves), and through pipe lined with soft rice paper (solid curves). Knowing that the naked mole-rats have a behavioral hearing threshold of 55 dB for 0.5 kHz (Fig. 1), and that they produce alarm calls with an average intensity of 86 dB at 20 cm, we estimate that an alarm call should be able to travel through the tunnel for about 15 m before call intensity drops below threshold.

A higher frequency of 3.1 kHz, roughly the frequency of peak energy for the signature calls, was attenuated by 8 dB (plain PVC pipe, open symbols) to 24 dB (pipe lined with soft rice paper, solid symbols) over 5 m. Hence, we estimate that signature calls, which are produced at 63 dB, should travel through the tunnel between 3 and 8 m before call intensity drops below the threshold of 50 dB. Higher frequency tones were attenuated more.

**Fig. 6:**
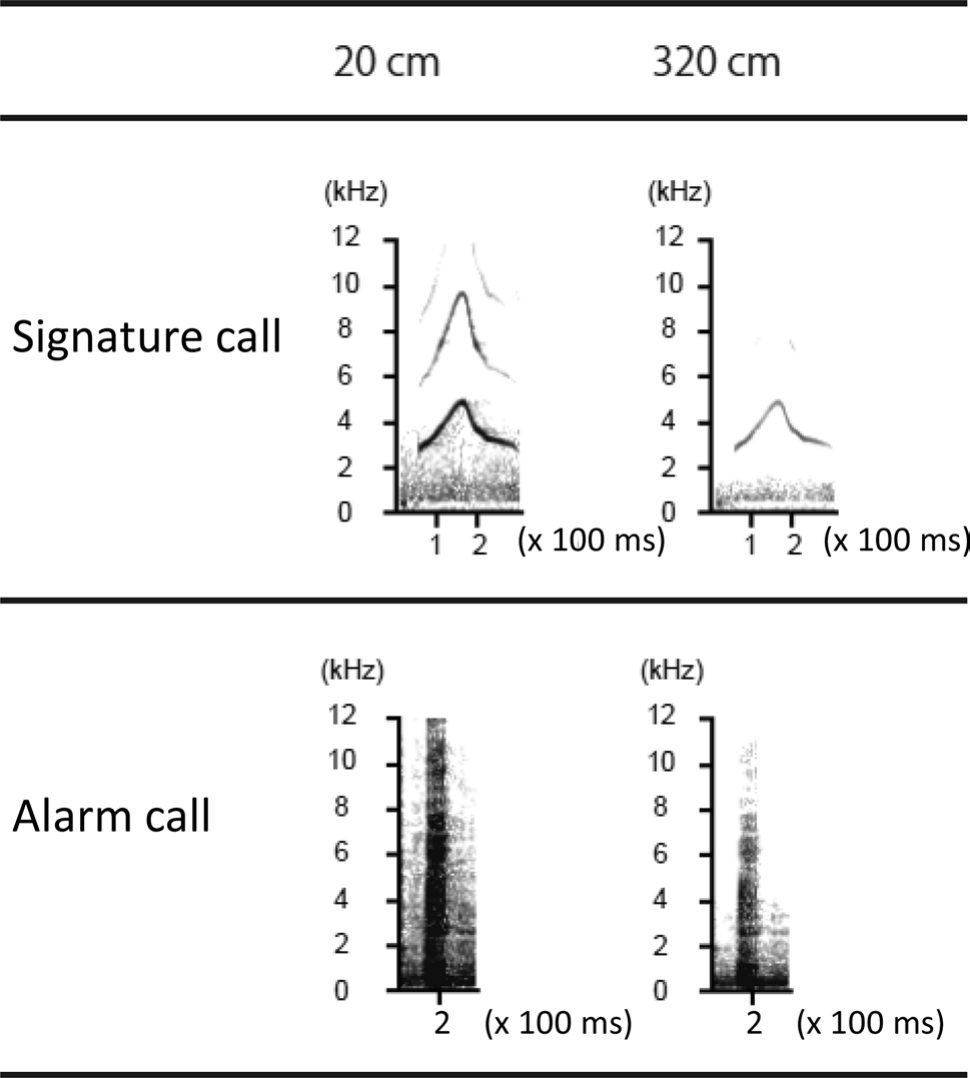
Spectrograms of a signature calls, and an alarm call after transmission through 0.2 and 3.2 m pipes

To determine how passing through the tubes would affect the spectral characteristics of calls, we played both call types through the pipes. Figure 6 shows the average power spectra of one representative alarm call and signature call after transmission through 3.2 m of pipe. Although the sound morphology was maintained for the alarm call, it was more degraded for the signature call.

## Discussion

Our main conclusion is that high intensity, low frequency alarm calls are audible over long distances through tunnels while lower intensity, higher frequency signature calls are audible over much shorter distances. This result is in agreement with basic acoustics. In both cases (alarm and signature calls), the distances that these calls propagate are well matched to their function. Also, even though the alarm and signature calls were substantially different from one another in spectral characteristics, both call types are well within the range of best hearing for the naked mole-rats.

### Propagation through tunnels

There are obvious drawbacks to measuring sound characteristics through PVC pipe instead of actual burrows in the field. Thus, while our conclusion that alarm calls propagate substantially farther than signature calls is well supported, our quantitative measures of attenuation through PVC pipes is likely an underestimate of what happens in actual burrows. However, data published by Lange et al. (2007) on sound propagation through actual burrows of Fukomys mole-rats is not that different from what we observed with the PVC pipe, particularly for low frequency sounds. For 0.5 kH, we found attenuation through the 4 cm pipe to be 2 dB/m. Lange et al. tested a similar frequency (0.4 kH) through burrows with an average diameter of 5 cm and found attenuation to be 3.1 dB/m. For higher frequencies (e.g. 3.1–3.2 kH), Lange et al. found considerably more attenuation through the 5 cm burrow (50.4 dB/m) compared to what we found with the PVC pipe (5 dB/m). We recognize that the difference between the findings of Lange et al. and the present report for a higher frequency are large but we do not have an explanation at the time to account for it. It is notable that attenuation measured by Lange et al. through the 5 cm burrow was the greatest they reported among eight burrows of various diameters and species. It is also notable that Lange et al. showed that curves in actual tunnels probably increase attenuation of sounds. Brett (1991) reported that naked mole-rat burrows have curves and branches. Another factor that would potentially increase attenuation in actual burrows would be the effect of colony mates blocking the burrows with their bodies. We also point out that while environmental background noise is low in burrows, the animals themselves could make substantial “noise” from vocalizing, digging, and moving about the burrows, which could potentially interfere with communication.

### Auditory thresholds

The ABR-derived measure of auditory sensitivity confirmed that the naked mole-rats have best hearing for low frequencies with high thresholds overall. These results are consistent with a previous, behaviorally derived audiogram for this species (Heffner and Heffner 1993). We also found that the amplitude of the ABR waveform was substantially smaller for the naked mole-rats compared to gerbils (and other mammals). We do not yet know why, or if, this is a common feature across subterranean mammals or particular to naked mole-rats.

Low frequency hearing appears to be a common characteristic of subterranean hearing, and low frequency vocalizations are common for these species as well (Bruns et al. 1988; Burda et al. 1992; Credner et al. 1997; Nevo 1999). This pattern has been reported for the Zambian mole-rat (Africa; Brückmann and Burda 1997; Credner et al. 1997), the blind mole-rat (Europe; Nevo et al. 1987; Heth et al. 1988; Bronchti et al. 1989), and the coruro (South America; Veitl et al. 2000; Begall et al. 2004).

A close relationship between tunnel propagation characteristics, hearing sensitivity, and call spectra may be common to other subterranean mammals. The blind mole-rat (*Spalax ehrenbergi*), a solitary-living species native to the Middle East, emits low frequency calls in the range of 0.5 kHz (Heth et al. 1986; Nevo et al. 1987). The range of most sensitive hearing for this species is 0.5–1.5 kHz (Bruns et al. 1988; Bronchti et al. 1989), and their tunnels show best propagation for frequencies around 0.5 kHz (Heth et al. 1986).

Despite the good match between best hearing sensitivity and call spectrum, the naked mole-rats’ overall high thresholds would greatly restrict the distance over which auditory-vocal communication would be effective in an open environment. However, naked mole-rats are never in an open environment. Rather, they live their entire lives within a tube-like burrow system that facilitates call propagation, much like a speaking tube on navy ships and playgrounds (Elliot and Foulkes 2011). There are a number of factors that affect sound propagation through tube-like structures, including temperature, humidity, and surface texture, but the most important factors are the diameter of the tube, and the frequency of the sound (Lange et al. 2007). Our measurements indicate that the low frequency alarm calls of naked mole-rats propagate well through tubes with a diameter based on naked mole-rat tunnels. Hence, the low background noise and the propagation characteristics of their tunnels, together with calls produced at high intensities, could compensate to a large extent for the naked mole-rats’ comparatively high auditory thresholds.

In contrast to alarm calls, signature calls, which are produced at higher frequencies and lower intensities, propagate over much shorter distances before becoming degraded and inaudible. However, this appears to be consistent with the behavioral context in which signature calls are used. Naked mole-rats consistently emit signature calls when touched by another mole-rat, suggesting a function in close contact communication.

### A coupled auditory-vocal communication system

A hallmark feature of acoustic communication systems across taxa is co-evolution of coupled signal production and auditory reception characteristics. Within their tunnels, the same phenomenon appears to apply to subterranean mammals. The interesting thing about these species is that the coupling takes place with higher auditory thresholds than are typical for the range of best hearing sensitivity of other mammals. Burda et al. (1992) have argued that the limiting factors for hearing sensitivity in subterranean mammals result from constraints on head size, and that given these constrains, middle ear structures are actually well adapted for low frequency, subterranean hearing. Furthermore, Lange et al. (2007) have suggested that decreased sensitivity in subterranean mammals may be a protective adaptation. Those authors measured attenuation of sound in burrows of Fukomys mole-rats in the field and they found that low frequencies not only propagated well, they could be substantially amplified.

Alternatively, in a closed tunnel system there may be less pressure to maintain the same high degree of auditory sensitivity typical of non-subterranean mammals. Because the naked mole-rats generate their calls at such relatively high intensities, it is interesting to speculate that the cost of maintaining high auditory sensitivity may outweigh the energetic cost of producing relatively high-intensity vocalizations. In this regard, another interesting feature of subterranean life that is related to high-intensity vocalizations is that the threat of predation based on predators hearing the communication calls is greatly reduced for subterraneans compared to surface dwelling rodents.
